# The Neural Association between Tendency to Forgive and Spontaneous Brain Activity in Healthy Young Adults

**DOI:** 10.3389/fnhum.2017.00561

**Published:** 2017-11-20

**Authors:** Haijiang Li, Jiamei Lu

**Affiliations:** Department of Psychology, Shanghai Normal University, Shanghai, China

**Keywords:** tendency to forgive, mentalizing, resting-state fMRI, ALFF, mPFC

## Abstract

The tendency to forgive (TTF) refers to one’s global dispositional level of forgiveness across situations and relationships. Previous brain imaging studies examined activation patterns underlying forgiving process, yet the association between individual differences in the TTF and spontaneous brain activity at resting-state remains unknown. In this study, resting-state functional magnetic resonance imaging (fMRI) was used to investigate the correlation between the TTF and spontaneous brain activity in a young adult sample. Participants were 178 young students (55 men) who completed the TTF scale and underwent a resting-state fMRI scan. Multiple regression analysis was conducted to assess the association between the regional amplitude of low-frequency fluctuations (ALFF) and TTF scores corrected for age and sex. Results showed that the ALFF value in the right dorsomedial prefrontal cortex (dmPFC), precuneus and inferior parietal lobule (IPL) were negatively associated with TTF scores. These findings suggest that the spontaneous brain activity of brain regions like the dmPFC, precuneus and IPL which are implicated in mentalizing and empathic response are associated with individual differences in the TTF.

## Introduction

Scientific investigation of forgiveness has a rapid increase from many perspectives within psychology in recent decades (Riek and Mania, 2012). Forgiveness is defined as a set of changing processes including reduced negative emotion toward the wrongdoers, decreased motivation to retaliate or punish, and increased benevolence toward the offender (Enright, 1991; McCullough et al., 2001; Beyens et al., 2015). There is still a lack of consensus on exact forgiveness definition (Hughes, 1997; McCullough et al., 2001; Riek and Mania, 2012), however, researchers would agree that forgiveness involved a prosocial changing process toward the transgressor (McCullough et al., 2001). The tendency to forgive (TTF) was described as one’s global dispositional level of forgiveness across situations and relationships (Brown, 2003). It measures individual differences in the tendency either to let go of one’s offense experiences or hold on to them. It only captures individual differences in the extent to which forgiveness typically occurs rather than the exact process of how or why it occurs (Brown, 2003). Previous studies found that individual differences in dispositional forgiveness not only has a negative association with mental disturbances (i.e., depression, rumination; Brown, 2003; Berry et al., 2005; McCullough et al., 2007), but also has a strong link to physical health outcomes (Toussaint and Cheadle, 2009; Lawler-Row et al., 2011).

Imaging studies investigating neural activation concerning forgiveness have found activity of the lateral prefrontal cortex (PFC), anterior cingulate cortex (ACC), medial PFC (mPFC), temporoparietal junction (TPJ) and precuneus which play a vital role in cognitive control and mentalizing (Will et al., 2015, 2016; Fatfouta et al., 2016; Billingsley and Losin, 2017). For example, forgiving judgments of one’s crime evoked elevated activation of superior frontal gyrus and precuneus (Farrow et al., 2001). This is supported by recent findings that increased activity of the dorsolateral PFC, inferior parietal lobule (IPL) and precuneus were observed when granting forgiveness toward the transgressor (Ricciardi et al., 2013). In addition, granting forgiveness was found to be correlated with participants’ self-reported perspective-taking and activation in brain regions concerning mentalizing (i.e., theory of mind) like dorsomedial prefrontal cortex (dmPFC) and TPJ, and in brain regions implicated in inhibit control, such as ACC and lateral PFC (Will et al., 2015). Researchers also found that when participants refrain from retaliation and act prosocially (i.e., forgive) toward offender will activate the dmPFC and functional connectivity between dmPFC and dorsal ACC negatively correlated to participants’ TTF (Fatfouta et al., 2016).

Although previous studies have examined activation patterns underlying granting forgiveness, the neural basis related to forgiveness is still unclear. Previous imaging studies mainly used indirect measures of forgiveness which are embedded within a particular method for inducing a transgression and manipulating forgiveness-related behaviors (Yamada et al., 2012; Beyens et al., 2015; Will et al., 2015). This kind of behavioral measures of forgiveness is thought to be weak in effect and examine different processes of forgiveness (Worthington et al., 2015). Thus, the dispositional measure of forgiveness can provide a relatively stable and global index of forgiveness. Combining with resting-state functional magnetic resonance imaging (fMRI), which do not need an externally prompted task, the present study will supplement previous neural findings based on different behavioral measures of forgiveness. Resting-state fMRI provides not only regional amplitude of low-frequency fluctuations (ALFF) but also data about resting-state functional connectivity (RSFC). Where ALFF analysis measures the intensity of regional spontaneous neuronal activity (Zang et al., 2007), RSFC detects the synchronization of spatially remote regions within a network and thus provides a complementary measure of network function (Biswal et al., 1995; Zang et al., 2007). Both ALFF and RSFC have been used to explore the neural bases of neurological and psychiatric disorders like schizophrenia (Hoptman et al., 2010; Dong et al., 2017), major depression disorders (Li et al., 2016; Zhong et al., 2017) and anxiety (Oathes et al., 2015; He et al., 2016). Spontaneous brain activity is also related to individual differences in personality (e.g., Yang et al., 2015; Kong et al., 2016), cognitive ability (Chen et al., 2016; Jung et al., 2016), and behavioral response tendency (Li et al., 2014) among healthy populations. These findings suggest that the ALFF and RSFC may reflect the potential neural mechanisms of the TTF.

In the current study, we aimed to explore the extent to which spontaneous brain activity (ALFF and RSFC) at resting-state was correlated to individual differences in the TTF. Based on previous imaging studies, we hypothesized that individual variations in TTF would be related to spontaneous brain activity differences in brain regions implicated in cognitive control and mentalizing, such as lateral PFC, mPFC, ACC, TPJ and precuneus.

## Materials and Methods

### Participants

Participants were 178 young adults (55 men, 139 women; mean age = 20.22, SD = 1.55, age range: 18–26 years) from Southwest University (SWU), Chongqing, China who volunteered as part of an ongoing project examining associations between brain imaging, creativity and mental health (Li et al., 2014; Wei et al., 2015; Liu et al., 2017). All participants were right-handed and physically healthy. None had a history of neurological or psychiatric illness assessed by a self-report questionnaire before scanning. This study was carried out in accordance with the recommendations of SWU Brain Imaging Center Institutional Ethics Review Board with written informed consent from all subjects. All subjects gave written informed consent in accordance with the Declaration of Helsinki. The protocol was approved by the SWU Brain Imaging Center Institutional Ethics Review Board. After completing all study protocols, participants were thanked for their time and received financial compensation.

### Tendency to Forgive Scale (TTF; Brown, 2003)

The TTF is a 4-item scale which assesses individual differences in the TTF one’s offence across situations and relationships (Brown, 2003). Sample items include, “I tend to get over it quickly when someone hurts my feelings” and “I have a tendency to harbor grudges”. Participants were asked to responsed on a five-point Likert scale that ranges from strongly disagrees (1) to strongly agree (5). The TTF has been demonstrated to have a reasonable internal reliability and high degree of stability over 8 weeks in prior study (Brown, 2003). The Chinese version of TTF were also shown to have a good levels of reliability and validity (Hu et al., 2005; Zhu, 2015). In this study, the TTF had an acceptable internal consistency, *α* = 0.57.

### MRI Data Acquisition

MR images were acquired on a 3T Siemens Trio MRI scanner (Siemens Medical, Erlangen, Germany). Participants were asked to remain still, close eyes and not fall sleep. Resting-state functional images were obtained using gradient-echo echo planar imaging (EPI) sequence with parameters: Slices = 32, TR/TE = 2000/30 ms, FA = 90°, FOV = 220 × 220 mm^2^, Thickness = 3 mm, slice gap = 1 mm, matrix = 64 × 64, resulting in a voxel with 3.4 × 3.4 × 4 mm^3^.

### Functional Imaging Data Preprocessing

The processing of the resting-state fMRI data were performed using statistical parametric mapping (SPM8^1^) and the Data Processing Assistant for Resting-State fMRI toolbox DPARSF (Yan and Zang, 2010). The first 10 volumes of the functional images were discarded because of signal equilibrium and adaptation of the participants to the scanning noise. Then, slice timing and head motion correction were conducted. Participant with head motion exceeding 3.0 mm maximum translation or 3° rotation was discarded from further analysis. Subsequently, registered images were spatially normalized to Montreal Neurological Institute (MNI) template (resampling voxel size = 3 × 3 × 3 mm^3^). After the spatial smoothing (full width at half maximum = 8 mm Gaussian kernel), linear trend of the time series was removed and a 0.01–0.1 Hz band-pass filter was applied.

### ALFF Calculation

Following previous calculation procedures (Zang et al., 2007), the preprocessed time series was transformed into the frequency domain in order to estimate the power spectrum for each voxel. The averaged square root of the power spectrum calculated within 0.01–0.1 Hz at each voxel was taken as ALFF. For standardization purposes, the ALFF of each voxel was divided by the global mean ALFF values within the gray matter mask.

### Resting-State Functional Connectivity Analysis

The regions (dmPFC, Precuneus and IPL, see “Results” section) that were found to be significantly associated with TTF scores were used as region of interest (ROI) and computed the RSFC between the seed ROI and all other voxels to examine the association between TTF and the functional connectivity between seed regions (i.e., mPFC, Precuneus and IPL) and other brain areas. The rationale was to first use the observed region(s) in which regional ALFF was significantly correlated with TTF scores as seed(s) to perform RSFC analyses and thus to map out the regions which were functionally connected with the seed as a network. Next, we examined whether the RSFC within this network correlated with TTF scores.

### Statistical Analysis

Statistical analyses of ALFF images and RSFC images were performed using SPM8. Multiple linear regression were used to explore the association between regional ALFF images and RSFC images and individual differences in TTF scores. The TFF scores were used as the variable of interest. Age and sex were entered as covariates of no interest.

All analyses were corrected for multiple comparisons using topological false discovery rate (FDR) correction (Chumbley et al., 2010). Overall significance was achieved when FDR-corrected threshold of *p* < 0.05 with an underlying voxel level threshold of *p* < 0.001, uncorrected.

## Results

### Tendency to Forgive (TTF) Scores

The mean TFF scores for the current sample was 13.26 (SD = 2.24) with a range of 7–19. No significant gender difference in TTF scores was found in the present study (*t*_(176)_ = 0.88, *p* = 0.34).

### Regional ALFF-Behavior analysis

**Figure 1:**
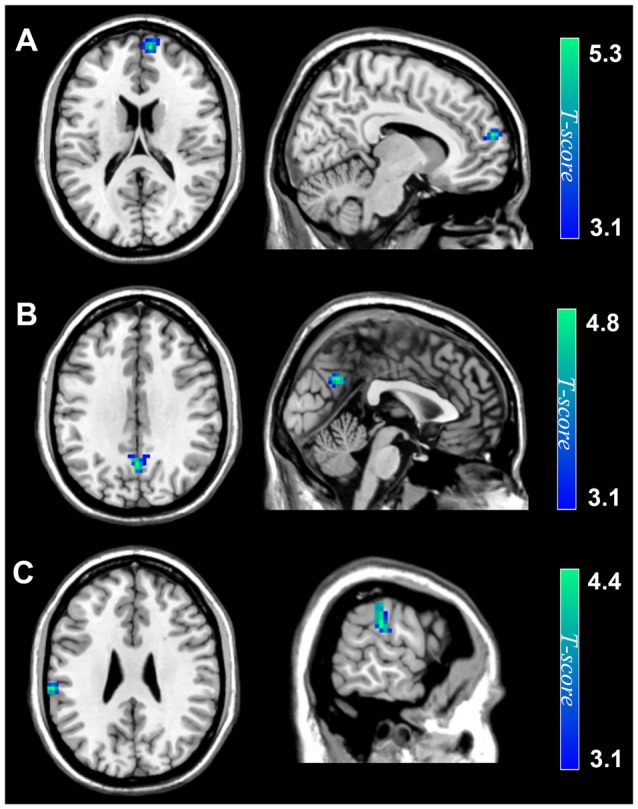
Spontaneous brain activity associations of TTF. Amplitude of low-frequency fluctuations (ALFF) of the dorsomedial prefrontal cortex (dmPFC) **(A)**, precuneus **(B)** and inferior parietal lobule (IPL) **(C)** was negatively correlated with TTF scores. Results are shown at *p* < 0.05, corrected for multiple comparisons at the cluster-level with topological false discovery rate (FDR) correction. The color density represents the *T*-score. TTF, tendency to forgive.

After controlling age and sex, TTF scores had significant and negative correlations with ALFF in the right dmPFC (peak MNI coordinate: 9, 60, 18; *t* = 5.24; Cluster size = 43 voxels, Table 1; Figure 1A), precuneus (peak MNI coordinate: 0, −60, 33; *t* = 4.75; Cluster size = 54, Table 1; Figure 1B) and left IPL (peak MNI coordinate: −63, −27, 27; *t* = 4.36; Cluster size = 45, Table 1; Figure 1C). No other significant relations were observed.

**Table 1:** Brain regions with significant association between regional ALFF and TTF scores.

Brain regions		MNI coordination			Cluster size (mm^3^)	Peak *T*-Value
	BA	*x*	*y*	*z*		
dmPFC	10	9	60	18	43	5.24
Precuneus	7	0	−60	33	54	4.75
IPL	40	−63	−27	27	45	4.36

*Note: ALFF, amplitude of low frequency; TTF, tendency to forgive; dmPFC, dorsomedial prefrontal cortex; IPL, inferior parietal lobule; BA, brodmann’s area*.

### RSFC-Behavior Analysis

This analyses did not find any significant associations between TTF scores and RSFC images seeded by mPFC, precuneus or TPJ after multiple comparison correction.

## Discussion

In the present study, we explored the neural functional substrates that underlies individual differences in the TTF using resting-state fMRI and TTF scale. Consistent with our hypotheses, the results showed that individual differences in the TTF was negatively correlated with ALFF value in dmPFC, precuneus and IPL. However, there is no significant relations between individual variations in TTF and RSFC seeded by dmPFC, precuneus or IPL after multiple comparison correction. These findings suggest that the spontaneous brain activity of brain regions that involved in mentalizing and empathic response may underlie the neural mechanism of individual differences in the TTF.

The current study found that the ALFF value variances in the dmPFC were negatively associated with individual differences in the TTF. The higher the ALLF value in dmPFC the lower an individual tend to forgive the transgression of others. The dmPFC is regarded as a mentalizing brain region, which enable us to infer the mental states (intentions, desires and beliefs), feelings, actions as well as more enduring dispositions of others (Van Overwalle, 2009; Baumgartner et al., 2012). It found that individual with the mPFC dysfunction were impaired in the function of mentalizing (Stuss et al., 2001), emotional regulation (Phan et al., 2004) and moral judgments (Koenigs et al., 2007). Common activations of dmPFC were also reported in response to forgive-related processing. For example, Will et al. (2015) investigated the neural correlations of forgiveness of initiators of social exclusion (i.e., excluders) and operationalized forgiveness as participants acting equitably toward participants who previously either excluded or included them. Results found that forgiving response was significantly related to self-reported perspective-taking and increased activation of dmPFC (Will et al., 2015), suggesting regions concerning mentalizing play a vital role in forgiving response. The same findings were replicated among participants experienced chronic peer rejection by the same group as well (Will et al., 2016). Evidence from parochial altruism study conducted by Baumgartner et al. (2012) found that in-group members was more forgivable than out-group members for committing the very same norm violation and this is associated with increased activation of dmPFC and connectivity between dmPFC and TPJ (regions that are involved in the mentalizing), as if participants tried to understand or justify in-group members’ behavior, as the authors noted (Baumgartner et al., 2012). Consistent with this, Fatfouta et al. (2016) found that participants were more likely to refrain from punishment (i.e., forgiveness) on unfair exchanges by a close partner relative to unknown person and the dmPFC was activated when the partner behaved unfairly relative to the unknown person. A recent study exploring forgiveness in the context of criminal sentences by asking participants who acted as jurors to consider reducing the sentences of defendants judged guilty of murder, using scenarios which can induce or not induce participants’ sympathy for the defendant. Imaging results found greater activation of dmPFC under the sympathy condition, and the activation intensity of dmPFC were significantly correlated to granting reduced sentences (i.e., forgiveness; Yamada et al., 2012). Additionally, researcher compared neural activation concerning forgivability judgments and empathic judgments and found that both empathic and forgivability judgments were related to significant activations of dmPFC (Farrow et al., 2001).

The current study also observed a negative association of precuneus ALFF value and individual differences in TTF. Precuneus is another key mentalizing region associated with inferring and representing mental states of others (Völlm et al., 2006; Lamm et al., 2011). One study found that both forgivability and empathic judgments on hypothetical crimes activated the region of precuneus (Farrow et al., 2001). In a study using imagined social scenarios conducted by Ricciardi et al. (2013), the precuneus activation was observed when participants link positive reappraisal and empathy toward an offender to forgive, suggesting empathic response toward transgressor is essential for forgiveness. In addition, forgiveness of hypothetical defendants under the condition of sympathy evoked greater precuneus activation as well (Yamada et al., 2012). These findings join recent review in suggesting that forgiveness recruits a wide array of mentalizing regions, including mPFC and precuneus (Billingsley and Losin, 2017). This was also supported by the theories of forgiveness therapy which assumed that empathic response toward offender after experienced transgression is one primary process for granting forgiveness (Baskin and Enright, 2004; Enright and Fitzgibbons, 2015). Thus, decreased spontaneous brain activity of mPFC and precuneus may facilitate participants with higher TTF scores inferring and representing mental states of others which may underlie individual differences in the TTF.

Decreased spontaneous brain activity in the IPL was observed in participants with higher TTF scores. Studies found that the IPL played a vital role in empathic response (Ruby and Decety, 2001). Dysfunction of IPL will cause an individual to be more emotional egocentricity because of disabling to perceive the emotions of others (Silani et al., 2013). Recent studies examined the association between neuropsychological indices of cerebral integrity and forgiveness in a population with traumatic brain injury (Johnstone et al., 2012, 2015) and found that decreased parietal lobe functions were negatively related to individual differences of forgiveness. Taking someone else’s perspective is an important prerequisite for understanding and predicting other people’s mental states like desires, thoughts or intentions. Take Ruby and Decety’s (2001) study for example, increased IPL activation was observed when participants simulated actions with a third-person perspective compared to the first-person perspective. Additionally, unforgiving responses (i.e., punishment) to participants who previously excluded them during a virtual ball-tossing game was related to increased activation in the IPL (Will et al., 2016). This was supported by a study which observed that forgiving judgment of an ambiguous offense evoked the activation of IPL (Strang et al., 2014). Moreover, compared to unforgiving the imagined agents who make them experienced emotionally hurt events, granting forgiveness toward offenders activated the IPL (Ricciardi et al., 2013). Thus, the association between the IPL spontaneous brain activity and TTF may suggest an important role of empathic response that IPL plays in granting forgiveness toward offender.

The main strength of the current study was the application of the resting-state fMRI exploring associations between spontaneous brain activity and the TTF. However, several limitations of this study should be mentioned. First, considering the cross-sectional research design, causal relations could not be drawn between the TTF and spontaneous brain activity. Hence, it is not clear whether spontaneous brain activity differences caused or resulted from a high level of TTF or whether causal associations are reciprocal. Longitudinal designs may be useful in exploring the status of spontaneous brain activity as predisposing factors that increase susceptibility for later changes in granting forgiveness. Second, caution is warranted in interpreting the results involving an high-order phenomenon of psychology like forgiveness. Only individual differences in the TTF which reflects one’s global dispositional level of forgiveness across situations and relationships was assessed. Whether the results consistent with the process of granting forgiveness prompt more studies to examine the associations between spontaneous brain activity and forgiving process. Third, the young adult sample drawn from a non-clinical setting in the current study may limit the extensibility of the present results to other age groups or general populations in which TTF scores and spontaneous brain activity are more normally-distributed. Four, negative correlation between TTF scores and spontaneous brain activity seems inconsistent with task-related fMRI studies during which increased activation of brain regions were found in the forgiveness condition. In fact, only values that are significantly greater than zero in ALFF images were used to calculate the association between forgiveness and spontaneous brain activity. This is a standardized procedure during data preprocessing. Thus, the negative correlation only means variation of ALFF values among each brain region is negatively related to the TTF. The exact relationship between negative correlation during spontaneous brain activity and activation in task-based fMRI need more studies to explore. Finally, findings should be considered provisional. While other spontaneous brain activity differences corresponding to varying of the TTF were not observed or did not survive after correction for multiple comparisons, replications in other samples are needed to demonstrate the reliability of findings across groups.

In summary, the present study examined for the first time the neural associations between spontaneous brain activity and individual differences in the TTF. Specifically, spontaneous brain activity variances in regions implicating in mentalizing and empathy like the dmPFC, precuneus and IPL were associated with individual differences in the TTF in a sample of healthy young adults.

## Author Contributions

HL and JL: conceived and designed the experiments. HL: collected and analyzed the data; contributed to the writing of the manuscript.

## Conflict of Interest Statement

The authors declare that the research was conducted in the absence of any commercial or financial relationships that could be construed as a potential conflict of interest.

## Footnotes

^1^http://www.fil.ion.ucl.ac.uk/spm
